# Long-Term Outcomes of a Multidisciplinary Treatment Strategy Using Neoadjuvant Gemcitabine Plus S-1 Based on Anatomical Resectability Classification and Lymph Node Status in Patients with Perihilar Cholangiocarcinoma

**DOI:** 10.3390/cancers18193238

**Published:** 2026-10-08

**Authors:** Naohisa Kuriyama, Shugo Mizuno, Yuki Segi, Haruna Komatusbara, Kazuyuki Gyoten, Takahiro Ito, Aoi Hayasaki, Takehiro Fujii, Yusuke Iizawa, Yasuhiro Murata, Akihiro Tanemura, Masashi Kishiwada

**Affiliations:** Department of Hepatobiliary Pancreatic and Transplant Surgery, Mie University Graduate School of Medicine, Tsu 5148507, Japan; mizunos@med.mie-u.ac.jp (S.M.); y-segi@med.mie-u.ac.jp (Y.S.); h-komatsu@med.mie-u.ac.jp (H.K.); k-gyoten@med.mie-u.ac.jp (K.G.); itotakahiro@med.mie-u.ac.jp (T.I.); a-hayasaki@med.mie-u.ac.jp (A.H.); t-fujii@med.mie-u.ac.jp (T.F.); uskm007@med.mie-u.ac.jp (Y.I.); yasumura@med.mie-u.ac.jp (Y.M.); iorichan@med.mie-u.ac.jp (A.T.); kishiwad@med.mie-u.ac.jp (M.K.)

**Keywords:** perihilar cholangiocarcinoma, neoadjuvant chemotherapy, gemcitabine, S-1, resectability classification, long-term survival

## Abstract

**Objectives:** The long-term impact of neoadjuvant chemotherapy (NAC) based on anatomical resectability for perihilar cholangiocarcinoma (PHC) remains unclear. We evaluated the mature long-term outcomes of our multidisciplinary treatment strategy based on anatomical resectability classification and regional lymph node (LN) status using gemcitabine plus S-1 (NAC-GS). **Methods:** Between September 2010 and August 2021, 110 consecutive PHC patients were classified as resectable (R; *n* = 47), borderline resectable (BR; *n* = 38), or unresectable locally advanced (UR-LA; *n* = 25) according to our anatomical resectability classification. NAC-GS was administered to patients with clinically positive LN metastasis and to BR and UR-LA patients. Long-term overall survival (OS), recurrence-free survival (RFS), and prognostic factors were evaluated. **Results:** NAC-GS was administered to 72 patients (65.5%). Curative-intent resection was achieved in 40 (85.1%), 25 (65.8%), and 6 (24.0%) patients in the R, BR, and UR-LA groups, respectively. In the overall cohort, anatomical resectability significantly stratified OS (*p* = 0.032). However, among patients who underwent curative-intent resection, OS and RFS were comparable across the three resectability groups. Those receiving NAC-GS showed favorable long-term survival despite having more advanced disease characteristics; however, this comparison was limited to selected patients who ultimately proceeded to surgery. Multivariable analysis identified preoperative serum carcinoembryonic antigen ≥5.5 ng/mL (HR: 3.174, *p* = 0.003), non-adjuvant therapy (HR: 2.222, *p* = 0.023) and pathological T4 (HR: 2.397, *p* = 0.037) as independent predictors of poor survival. **Conclusions:** Anatomical resectability was associated with long-term prognosis and the likelihood of achieving curative-intent resection. Selected patients who ultimately underwent curative-intent resection after NAC-GS showed favorable long-term outcomes; however, these findings do not establish a survival benefit of NAC-GS over upfront surgery. Prospective multi-institutional validation is warranted.

## Simple Summary

Perihilar cholangiocarcinoma is a challenging cancer for which surgery offers the best chance of long-term survival, but many patients have locally advanced disease at diagnosis. We evaluated the long-term outcomes of 110 patients treated using a multidisciplinary strategy based on anatomical resectability classification and regional lymph node status. Neoadjuvant chemotherapy with gemcitabine plus S-1 was selectively administered to patients with more advanced disease before surgery. Anatomical resectability was associated with the likelihood of achieving curative-intent resection and with survival in the overall population. However, among patients who underwent curative-intent resection, long-term survival did not significantly differ according to their initial resectability classification. These findings support a multidisciplinary treatment strategy incorporating neoadjuvant chemotherapy and highlight the need for more effective neoadjuvant treatments for patients with biologically aggressive disease.

## 1. Introduction

Perihilar cholangiocarcinoma (PHC) remains one of the most challenging malignancies in hepatobiliary surgery because of its complex anatomical location, frequent vascular invasion, and high incidence of locoregional and distant recurrence even after curative-intent resection. Although major hepatectomy combined with biliary resection and reconstruction is the only potentially curative treatment, long-term survival remains unsatisfactory because of the aggressive biological behavior of the disease and the high risk of postoperative recurrence [1,2].

Recent advances in systemic therapy have substantially changed the treatment strategy for biliary tract cancer (BTC). The BILCAP trial demonstrated the benefit of adjuvant capecitabine after curative resection and established adjuvant chemotherapy (AC) as a standard treatment in Western countries [3]. Subsequently, the Japanese phase III JCOG1202 (ASCOT) trial demonstrated that adjuvant S-1 significantly improved overall survival compared with observation, establishing S-1 as the standard AC for resected BTC in Japan [4]. Furthermore, the TOPAZ-1 and KEYNOTE-966 trials have established immunochemotherapy as the first-line treatment for advanced BTC [5,6]. As perioperative treatment has evolved, increasing attention has been directed toward neoadjuvant chemotherapy (NAC) for patients with anatomically advanced BTC.

To date, evidence supporting neoadjuvant therapy for PHC remains limited. Most published studies have been small retrospective series with heterogeneous patient populations and treatment regimens, and high-level evidence from randomized controlled trials is still lacking [7,8]. Matsuyama et al. reported encouraging outcomes of gemcitabine plus S-1 (GS)-based NAC for patients with borderline resectable PHC according to their institutional resectability criteria, supporting the potential role of NAC in selected patients [9]. Nevertheless, whether a treatment strategy based on standardized anatomical resectability criteria provides durable survival remains unknown.

To optimize treatment selection, we established an anatomical resectability classification for PHC in 2010 based on the longitudinal extent of biliary invasion and vascular involvement assessed by preoperative imaging. Patients were classified into resectable (R), borderline resectable (BR), and unresectable locally advanced (UR-LA) categories. Based on this classification together with clinical lymph node (LN) status, we introduced a multidisciplinary treatment strategy in which NAC-GS was administered to patients with BR or UR-LA disease and those with clinically positive LN metastasis. We previously demonstrated the feasibility of this strategy and subsequently reported favorable perioperative and mid-term oncological outcomes [10,11].

Importantly, the chemotherapy regimen itself has continued to evolve. At our institution, the standard NAC regimen has recently been updated from GS to gemcitabine, cisplatin, and S-1 (GCS), while the multicenter randomized phase III JCOG1920 (NABICAT) trial was initiated to evaluate GCS followed by surgery versus surgery-first for patients with resectable BTC, reflecting the growing interest in perioperative systemic therapy for BTC. Therefore, validation of the treatment strategy itself, rather than a specific chemotherapy regimen, has become increasingly important as perioperative systemic therapy continues to evolve. The present study represents the mature long-term analysis of our institutional experience accumulated over more than a decade. We aimed to evaluate the long-term outcomes of a multidisciplinary treatment strategy based on anatomical resectability classification and clinical LN status using NAC-GS in patients with PHC and to identify the remaining challenges for future neoadjuvant treatment strategies.

## 2. Materials and Methods

### 2.1. Study Design and Patients

This retrospective cohort study evaluated the long-term outcomes of a multidisciplinary treatment strategy for patients with PHC implemented at Mie University Hospital. Patients were enrolled between September 2010 and August 2021, and follow-up was continued until 31 July 2026.

The diagnosis of PHC was confirmed by cytological analysis of bile juice or histological analysis of biopsy specimens obtained using endoscopic retrograde cholangiography. Patients with evident distant metastasis at the time of enrollment were excluded. During the study period, 110 consecutive patients were enrolled and analyzed.

For the present analysis, the primary endpoint was overall survival (OS). Secondary endpoints included the rate of curative-intent resection according to the initial anatomical resectability classification, recurrence-free survival (RFS) after curative-intent resection, perioperative outcomes, prognostic factors for OS after resection, and pathological response to NAC-GS.

### 2.2. Anatomical Resectability Classification

Our institutional anatomical resectability classification has been described previously [10,11]. This classification is based exclusively on anatomical tumor extension and does not incorporate biological or conditional factors. Briefly, resectability was determined using preoperative multidetector-row computed tomography, magnetic resonance imaging, and cholangiography based on two anatomical factors: biliary extension and vascular involvement (Table 1).

The biliary factor was defined according to whether R0 resection could be achieved by right or left trisectionectomy or a less extensive hepatectomy. The vascular factor was defined according to the feasibility of portal vein (PV) and/or hepatic artery (HA) resection and reconstruction.

Patients were classified into three categories as follows:

R: R0 resection is achievable by right or left trisectionectomy or a less extensive hepatectomy without the need for PV and/or HA resection and reconstruction.

BR: R0 resection is achievable by right or left trisectionectomy or a less extensive hepatectomy, but combined PV and/or HA resection with vascular reconstruction is required and technically feasible.

UR-LA: R0 resection is not achievable even with right or left trisectionectomy, and/or combined PV and/or HA resection with safe vascular reconstruction is technically infeasible.

Importantly, the UR-LA category indicates anatomical unresectability at the time of initial diagnosis and does not necessarily imply permanent unresectability. Patients initially classified as UR-LA were therefore eligible for reassessment of resectability after NAC. Patients initially classified as UR-LA who subsequently underwent curative-intent resection after NAC were considered to have undergone conversion surgery.

Initial anatomical resectability classification and post-NAC reassessment were performed by consensus at a multidisciplinary conference involving hepatobiliary surgeons and radiologists. Because these assessments were performed as part of clinical decision-making, the assessors were not blinded to the patients’ clinical information or treatment course. Formal assessment of interobserver agreement was not performed.

### 2.3. Treatment Strategy

**Figure 1:**
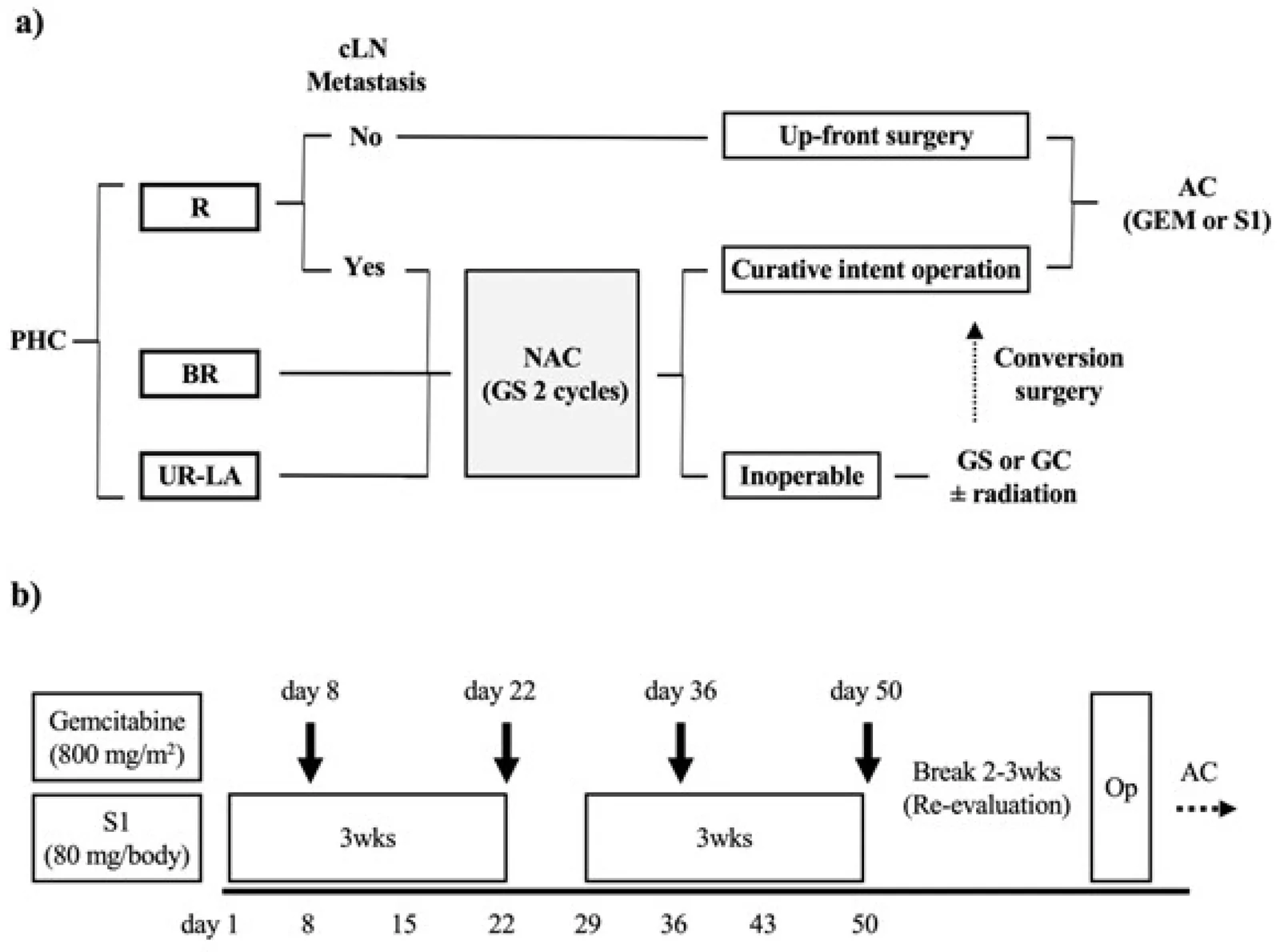
Institutional treatment algorithm for perihilar cholangiocarcinoma and protocol of 2 cycles Gemcitabine plus S-1 therapy. (**a**) Our institutional treatment algorithm for PHC, (**b**) Protocol of NAC-GS. PHC: perihilar cholangiocarcinoma, R: resectable, BR: borderline resectable, UR-LA: unresectable locally advanced, LN: lymph node, NAC: neoadjuvant chemotherapy, GS: gemcitabine plus S-1, GC: gemcitabine plus cisplatin, AC: adjuvant chemotherapy, GEM: gemcitabine, op: operation.

The institutional treatment algorithm is shown in Figure 1a. All treatment decisions were made according to a predefined institutional treatment algorithm through multidisciplinary discussion involving hepatobiliary surgeons and radiologists.

Patients classified as BR or UR-LA and those with clinically positive regional LN metastasis received NAC unless contraindicated because of poor performance status or impaired liver function. Clinical regional LN metastasis for treatment allocation was defined as positive FDG uptake in regional LNs on FDG-PET. CT and MRI were used for anatomical staging and assessment of tumor extent; however, morphological LN findings on CT or MRI alone were not used to define clinical LN positivity in the treatment algorithm.

The standard neoadjuvant treatment consisted of two cycles of gemcitabine plus S-1 (GS) in Figure 1b. After completion of the initial two cycles, treatment response and anatomical resectability were reassessed at a multidisciplinary conference using updated imaging studies. Curative-intent surgery, including conversion surgery for patients initially classified as UR-LA, was considered when R0 resection was judged potentially achievable by the planned hepatectomy with or without portal vein and/or hepatic artery resection and reconstruction, no distant metastasis was identified, and adequate future remnant liver function and general condition for the planned surgery were confirmed. However, precise radiological reassessment of local tumor extension after NAC was occasionally difficult because biliary stents had already been placed in many patients. Therefore, in selected patients without definite contraindications to resection, surgical exploration was undertaken, and final resectability was sometimes determined based on intraoperative findings. When additional local disease control was considered beneficial, further preoperative treatment, including additional systemic chemotherapy and/or radiotherapy, was individualized according to tumor response, anatomical resectability, and the patient’s general condition.

The primary objective of NAC was not only tumor downstaging but also optimization of patient selection for curative-intent surgery through repeated multidisciplinary assessment. Patients classified as R without clinical LN metastasis generally underwent upfront surgery.

### 2.4. Biliary Drainage Management

Preoperative biliary drainage was performed when clinically indicated, primarily targeting the future remnant liver. Until 2016, endoscopic biliary drainage was mainly performed using conventional plastic stents placed across the sphincter of Oddi. From approximately 2017, inside plastic stents placed above the sphincter of Oddi were preferentially used to reduce duodenobiliary reflux and the risk of recurrent biliary obstruction and cholangitis [12,13]. The detailed technique and clinical outcomes of inside stent placement have been reported previously [14].

During NAC, patients were closely monitored for biliary obstruction and cholangitis. When recurrent cholangitis occurred, biliary drainage was reassessed and additional endoscopic interventions were performed as clinically indicated, including bilateral inside stent placement or additional metallic stent placement. NAC was temporarily interrupted when necessary until cholangitis was adequately controlled.

### 2.5. Neoadjuvant Chemotherapy

Gemcitabine (800 mg/m^2^) was administered intravenously on days 1 and 21, and S-1 was administered orally at a fixed dose of 80 mg/body/day, divided into two daily doses, on days 1–21 of a 28-day cycle. Two cycles were planned as the standard neoadjuvant treatment (Figure 1b).

Treatment-related adverse events were graded according to the National Cancer Institute Common Terminology Criteria for Adverse Events (CTCAE), version applicable at the time of treatment.

### 2.6. Surgical Indication and Procedures

The indications for curative-intent surgery and the surgical procedures were determined according to our previously reported institutional treatment strategy [10].

Briefly, the extent of hepatectomy was selected according to the longitudinal extent of biliary invasion and vascular involvement to achieve R0 resection while preserving adequate future remnant liver. Future remnant liver function was evaluated using three-dimensional computed tomography volumetry combined with 99mTc-galactosyl human serum albumin (99mTc-GSA) scintigraphy. Functional remnant liver reserve was assessed using the functional remnant indocyanine green clearance (functional rem-KICG), calculated from the functional remnant liver ratio and the preoperative KICG. Major hepatectomy was considered feasible when the functional rem-KICG was ≥0.05, as previously reported [15]. PV embolization was performed when the future remnant liver volume was considered insufficient.

Vascular resection and reconstruction were performed when technically feasible and necessary to achieve negative surgical margins. Curative-intent surgery was performed when R0 resection was considered achievable in the absence of distant metastasis and with adequate remnant liver function, although the final decision occasionally required intraoperative assessment because the extent of biliary and vascular invasion could not always be accurately evaluated after biliary drainage.

Further details of our surgical strategy and techniques for PHC, including the transhepatic hilar approach and combined vascular resection and reconstruction, have been described previously [16].

### 2.7. Postoperative Complications

Postoperative complications were graded according to the Clavien–Dindo classification [17], and major postoperative complications were defined as Clavien–Dindo grade III or higher. Bile leakage, post-hepatectomy liver failure (PHLF), and post-hepatectomy hemorrhage were independently defined and graded according to the corresponding criteria of the International Study Group of Liver Surgery (ISGLS) [18,19,20]. Postoperative pancreatic fistula (POPF) was defined and graded according to the 2016 International Study Group on Pancreatic Surgery (ISGPS) criteria [21]. Intra-abdominal abscess was defined as a postoperative intra-abdominal infected fluid collection requiring percutaneous or surgical drainage, including placement of a new drainage tube or exchange of an existing drainage tube. Postoperative mortality was defined as death within 90 days after surgery.

### 2.8. Pathological Assessment

Histopathological findings were staged according to the American Joint Committee on Cancer (AJCC) Cancer Staging Manual, 8th edition [22]. Pathological T4 disease was defined as tumor invasion of the main portal vein or its branches bilaterally, the common hepatic artery, or unilateral second-order biliary radicals with contralateral portal vein or hepatic artery involvement. R1 status was defined based on microscopic tumor exposure at any biliary, vascular, and hepatic parenchyma resection margin of the surgical specimen. R2 status was also defined based on macroscopic tumor exposure or distant metastasis including intrahepatic metastasis.

Pathological response to NAC was evaluated by pathologists specializing in hepatobiliary pathology who were blinded to the patients’ clinical outcomes. The response was assessed based on the proportion of residual viable tumor cells in the primary tumor. Patients with <30% residual viable tumor cells were classified as high responders, whereas those with ≥30% residual viable tumor cells were classified as low responders. Regional lymph nodes and resection margins were not included in the assessment of pathological response. The 30% cutoff was used for the exploratory analysis in the present study and was not considered an established or universally validated cutoff for pathological response in PHC.

### 2.9. Postoperative Chemotherapy

Postoperative adjuvant chemotherapy was recommended for all patients who underwent curative-intent resection and was initiated 4–8 weeks after surgery whenever clinically feasible. During the study period, gemcitabine (800 mg/m^2^ every 2 weeks) was initially used as the standard adjuvant regimen. Since June 2013, S-1 (60 mg/m^2^/day, orally twice daily on days 1–28 of a 42-day cycle) became the preferred regimen according to our institutional treatment policy. Adjuvant chemotherapy was continued for at least 6 months whenever tolerated. When treatment-related toxicity occurred, the regimen was modified at the discretion of the treating physician.

### 2.10. Postoperative Follow-Up

Patients were followed at regular outpatient visits after surgery. Laboratory tests, including liver function tests and serum tumor markers, were performed every 2–3 months. Four-phase contrast-enhanced multidetector-row computed tomography was performed every 4 months during the first 2 postoperative years and every 6 months thereafter. Additional imaging studies were performed whenever recurrence was clinically suspected, including cases with increasing serum tumor marker levels.

### 2.11. Statistical Analysis

Continuous variables are presented as the median (range) and were compared using the Mann–Whitney U test. Categorical variables were compared using the χ^2^ test or Fisher’s exact test, as appropriate. Overall survival (OS) was defined as the interval from initiation of treatment to death from any cause or the last follow-up. Disease-specific survival (DSS) was defined as the interval from initiation of treatment to death attributable to PHC. Recurrence-free survival (RFS) was calculated from the date of curative-intent surgery to the date of first documented recurrence or death from any cause, whichever occurred first. Patients alive without recurrence at the last follow-up were censored. Patients who were alive at the last follow-up or who died from causes unrelated to PHC were censored for the DSS analysis. Survival curves were estimated using the Kaplan–Meier method and compared using the log-rank test. The final follow-up date was 31 July 2026. To identify prognostic factors after resection, univariable and multivariable analyses were performed using the Cox proportional hazards regression model. Variables with a *p* value < 0.10 in the univariable analysis were entered into the multivariable model. Hazard ratios (HRs) with 95% confidence intervals (CIs) were calculated. The optimal cutoff value for preoperative serum CEA was determined using Cutoff Finder [23] based on overall survival. The cutoff value showing the most significant split by the log-rank test was selected, resulting in a cutoff value of 5.5 ng/mL.

All statistical analyses were performed using IBM SPSS Statistics for Mac, version 29.0 (IBM Corp., Armonk, NY, USA). All tests were two-sided, and a *p* value < 0.05 was considered statistically significant.

## 3. Results

### 3.1. Patient Characteristics

**Figure 2:**
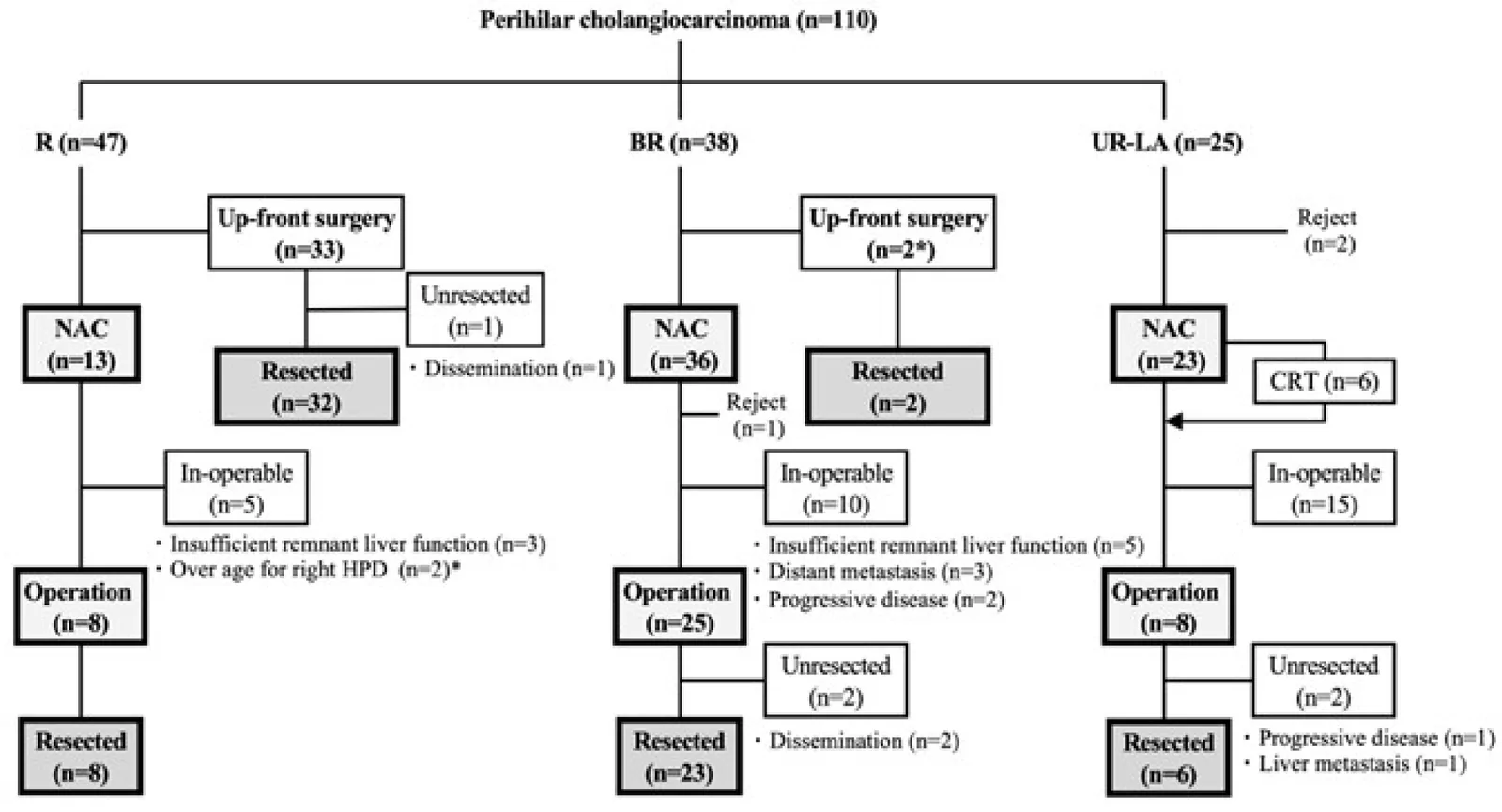
Study flow diagram for perihilar cholangiocarcinoma. R: resectable, BR: borderline resectable, UR-LA: unresectable locally advanced, NAC: neoadjuvant chemotherapy, CRT: chemoradiation therapy, HPD: hepatopancreatoduodenectomy, *: Patients aged 80 or older are not eligible for right HPD.

Between September 2010 and August 2021, 110 consecutive patients with localized PHC who met the study criteria were enrolled. According to our institutional anatomical resectability classification, 47 patients (42.7%) were classified as R, 38 (34.5%) as BR, and 25 (22.7%) as UR-LA. Baseline patient characteristics and study flow diagram are summarized in Table 2 and Figure 2.

There were no significant differences among the three groups regarding age, sex, biliary drainage, neutrophil-to-lymphocyte ratio, prognostic nutritional index, serum carcinoembryonic antigen (CEA) and carbohydrate antigen (CA19-9) level. However, patients in the BR and UR-LA groups more frequently had clinical T4 tumors and FDG-PET-positive regional LN metastasis than those in the R group (*p* < 0.001 and *p* = 0.038, respectively). NAC-GS was administered to 72 patients (65.5%), including 13 patients (27.7%) in the R group, 36 (94.7%) in the BR group, and 23 (92.0%) in the UR-LA group (*p* < 0.001). More than 84% of patients in each group completed the planned initial two cycles. The median relative dose intensity was maintained at 100% for S-1 and exceeded 83% for gemcitabine. Grade 3 or 4 adverse events occurred in 26 patients (36.1%), with grade 3 cholangitis being the most frequent severe toxicity. Curative-intent resection was achieved in 40 patients (85.1%) in the R group, 25 (65.8%) in the BR group, and 6 (24.0%) in the UR-LA group (*p* < 0.001).

Among the 47 patients classified as R, 13 received NAC. Of these, 8 underwent curative-intent operation, whereas 5 did not undergo operation because of insufficient remnant liver function (*n* = 3) or advanced age (≥80 years) precluding hepatopancreatoduodenectomy (HPD) (*n* = 2).

Among the 38 patients classified as BR, 36 received NAC, whereas 2 underwent upfront surgery because persistent cholangitis that was difficult to control precluded the safe administration of NAC. Among the 36 patients who received NAC, 1 declined surgery after NAC. Twenty-five patients underwent surgical exploration; however, peritoneal dissemination was identified intraoperatively in 2 patients, precluding curative-intent operation. The remaining 10 patients did not undergo surgery because of insufficient remnant liver function (*n* = 5), development of distant metastases (*n* = 3), or local progressive disease (*n* = 2).

Among the 25 patients classified as UR-LA, 2 declined treatment before initiation of NAC, and the remaining 23 received NAC. Eight patients subsequently underwent surgical exploration; however, curative-intent resection was not performed in 2 patients because of intraoperatively confirmed local progressive disease (*n* = 1) and liver metastasis (*n* = 1). Consequently, 6 patients underwent conversion surgery with curative intent. The remaining 15 patients did not undergo operation because of local progressive disease (*n* = 8), insufficient remnant liver function (*n* = 2), development of distant metastases (*n* = 2), or deterioration of performance status (*n* = 3).

### 3.2. Survival According to Anatomical Resectability Classification

**Figure 3:**
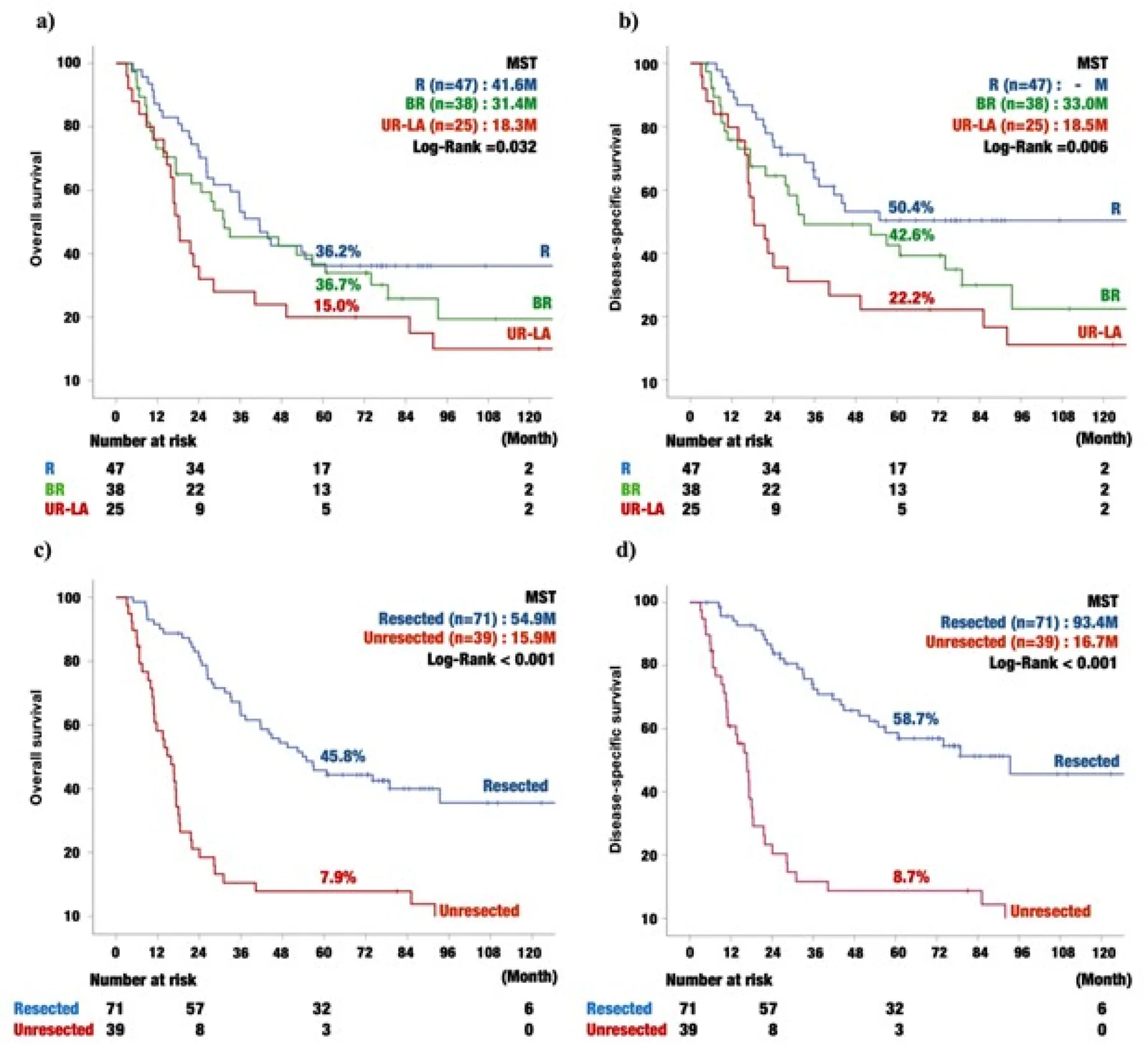
Overall survival and disease-specific survival curves in the 110 patients with perihilar cholangiocarcinoma. Comparison of (**a**) overall survival and (**b**) disease-specific survival according to anatomical resectability classification. Comparison of (**c**) overall survival and (**d**) disease-specific survival between resected and unresected patients. R: resectable, BR: borderline resectable, UR-LA: unresectable locally advanced, MST: median survival time.

The median follow-up period for survivors was 78.9 months. The Kaplan–Meier survival curves according to anatomical resectability classification are shown in Figure 3a,b. The median OS was 41.6 months in the R group, 31.4 months in the BR group, and 18.3 months in the UR-LA group. The corresponding 5-year OS rates were 36.2%, 36.7%, and 15.0%, respectively. OS differed significantly among the three groups (*p* = 0.032).

Similarly, DSS was significantly stratified according to anatomical resectability classification (*p* = 0.006). The median DSS was not reached in the R group, 33.0 months in the BR group, and 18.5 months in the UR-LA group, with corresponding 5-year DSS rates of 50.4%, 42.6%, and 22.2%, respectively (Figure 3b).

### 3.3. Survival According to Resection Status

The survival outcomes according to resection status are shown in Figure 3c,d. Patients who underwent curative-intent resection had significantly better survival than those who did not undergo resection. The median OS was 54.9 months in the resected group and 15.9 months in the unresected group, with corresponding 5-year OS rates of 45.8% and 7.9%, respectively (*p* < 0.001).

Likewise, the median DSS was 93.4 months in the resected group and 16.7 months in the unresected group, with 5-year DSS rates of 58.7% and 8.7%, respectively (*p* < 0.001) (Figure 3d). These findings indicate that long-term survival was achieved almost exclusively in patients who underwent curative-intent resection.

### 3.4. Surgical Outcomes of Resected Patients

The clinicopathological characteristics and perioperative outcomes of the 71 patients who underwent curative-intent resection are summarized in Table 3. Combined vascular resection was performed significantly more frequently in patients initially classified as BR or UR-LA than in those classified as R (76.0%, 66.7%, and 10.0%, respectively; *p* < 0.001). Despite the greater surgical complexity in the BR and UR-LA groups, the incidence of major postoperative complications (Clavien–Dindo grade ≥III) did not differ significantly among the R, BR, and UR-LA groups (52.5%, 52.0%, and 50.0%, respectively; *p* = 0.993). No significant differences were observed among the three groups in the incidence of ISGLS grade B/C bile leakage, PHLF, post-hepatectomy hemorrhage, clinically relevant POPF, intra-abdominal abscess requiring drainage, reoperation, or postoperative hospital stay. One 90-day postoperative death occurred in the BR group. The R0 resection rates were 72.5%, 64.0%, and 66.7% in the R, BR, and UR-LA groups, respectively, with no significant difference among the groups (*p* = 0.743).

### 3.5. Survival After Curative-Intent Resection

**Figure 4:**
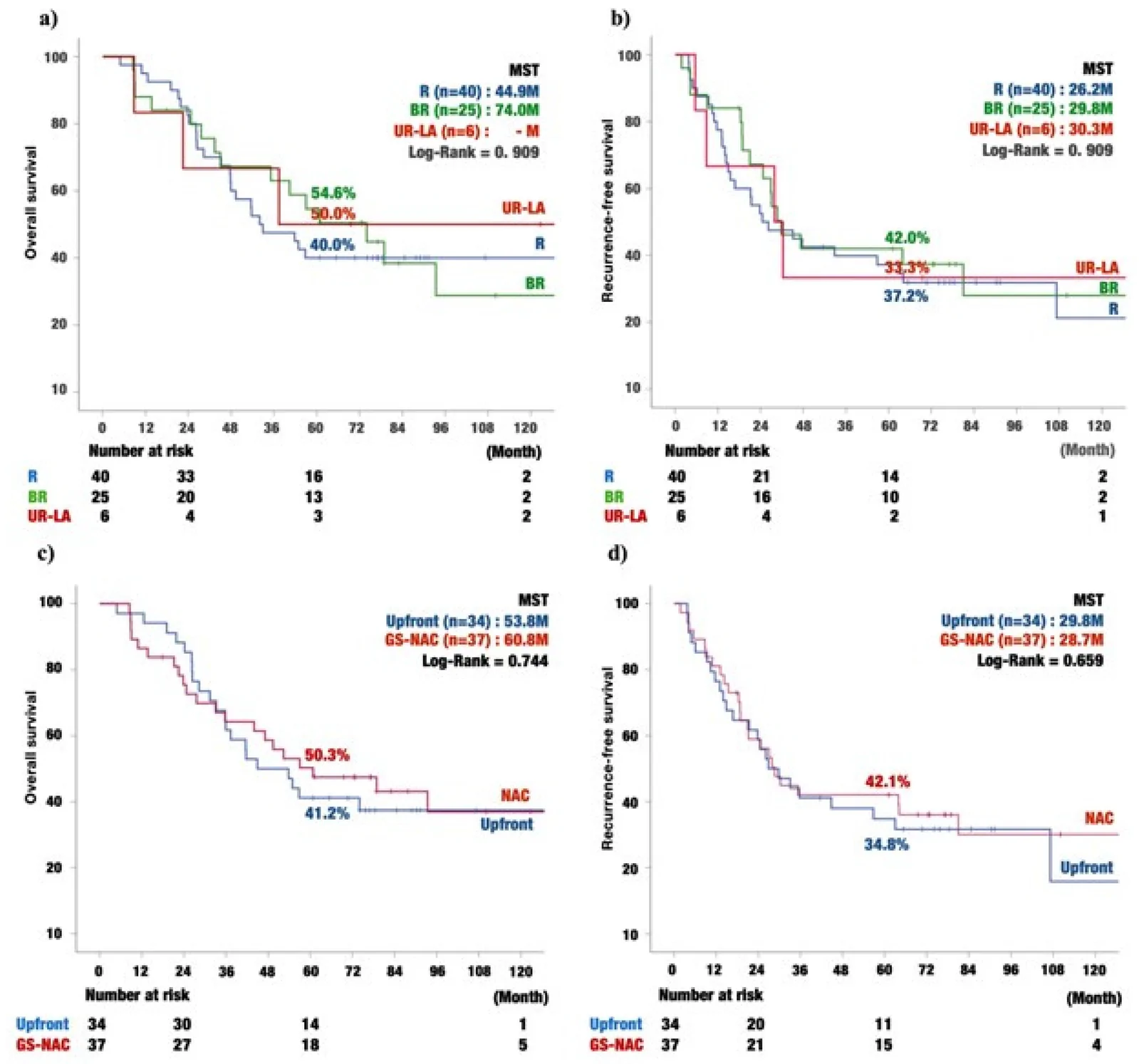
Overall survival and recurrence-free survival curves in the resected 71 patients with perihilar cholangiocarcinoma. Comparison of (**a**) overall survival and (**b**) recurrence-free survival according to anatomical resectability classification. Comparison of (**c**) overall survival and (**d**) recurrence-free survival between patients who underwent upfront surgery and NAC-GS followed by curative-intent resection. R: resectable, BR: borderline resectable, UR-LA: unresectable locally advanced, MST: median survival time, NAC: neoadjuvant chemotherapy, GS: gemcitabine plus S-1.

Survival analyses of the 71 resected patients are shown in Figure 4a,b. In contrast to the survival analysis of the entire cohort, anatomical resectability classification no longer influenced postoperative prognosis after curative-intent resection. The median OS was 44.9 months in the R group, 74.0 months in the BR group, and was not reached in the UR-LA group, with corresponding 5-year OS rates of 40.0%, 54.6%, and 50.0%, respectively (*p* = 0.909).

Similarly, RFS did not differ among the three groups. The median RFS was 26.2 months, 29.8 months, and 30.3 months in the R, BR, and UR-LA groups, respectively, and the corresponding 5-year RFS rates were 37.2%, 42.0%, and 33.3% (*p* = 0.909).

These findings indicate that once curative-intent resection was achieved, long-term survival was comparable regardless of the initial anatomical resectability classification.

### 3.6. Survival According to Neoadjuvant Chemotherapy

To evaluate the impact of NAC-GS, survival outcomes were compared between patients who underwent upfront surgery and those who underwent surgery following NAC-GS (Figure 4c,d). Clinicopathological characteristics of the resected patients according to treatment strategy are summarized in Table 4. Patients in the NAC group more frequently required combined vascular resection and reconstruction and tended to have a higher incidence of pathological T4 disease than those who underwent upfront surgery, indicating that the NAC group included more anatomically advanced tumors.

Despite these differences, no significant differences were observed in either OS or RFS between the two groups. The median OS was 53.8 months in the upfront surgery group and 60.8 months in the NAC group, with corresponding 5-year OS rates of 41.2% and 50.3%, respectively (*p* = 0.744). Likewise, the median RFS was 29.8 months and 28.7 months, with corresponding 5-year RFS rates of 42.1% and 34.8%, respectively (*p* = 0.659).

### 3.7. Prognostic Factors After Curative-Intent Resection

The results of the univariable and multivariable analyses are summarized in Table 5. On univariable analysis, elevated preoperative serum CEA level, pathological T4 disease, and several pathological factors were associated with poorer OS. Multivariable Cox regression analysis identified only preoperative serum CEA ≥ 5.5 ng/mL (HR: 3.174, *p* = 0.003), non-adjuvant therapy (HR: 2.222, *p* = 0.023) and pathological T4 disease (HR: 2.397, *p* = 0.037) as independent predictors of poor overall survival.

### 3.8. Histological Response to Neoadjuvant Chemotherapy

Among the 33 patients who completed NAC-GS and subsequently underwent curative-intent resection, pathological response was evaluated according to the proportion of viable cancer cells in the resected specimen (Supplementary Figure S1).

Thirteen patients (39.4%) were classified as high responders (<30% viable tumor cells), whereas 20 (60.6%) were classified as low responders (≥30% viable tumor cells). Clinicopathological characteristics according to histological response are summarized in Supplementary Table S1. Although no statistically significant differences in OS or RFS were observed between the two groups, high responders showed a numerical trend toward more favorable long-term outcomes than low responders (Supplementary Figure S2). Given the limited sample size, these findings should be considered exploratory (Supplementary Figure S2).

## 4. Discussion

The present study represents, to our knowledge, the first report demonstrating mature long-term outcomes of a multidisciplinary treatment strategy based on anatomical resectability classification and regional LN status using NAC-GS in patients with PHC. The principal findings of this study were fourfold. First, the anatomical resectability classification successfully stratified long-term survival in the overall cohort. Second, once curative-intent resection was achieved, long-term survival became comparable regardless of the initial anatomical resectability classification. Third, selected patients who received NAC-GS and subsequently underwent curative-intent operation achieved favorable long-term survival despite having more anatomically advanced disease at baseline. Finally, elevated preoperative serum CEA and pathological T4 disease remained independent predictors of poor survival after resection, indicating that further improvement in systemic therapy is required.

The concept of resectability classification has become well established in pancreatic cancer, where anatomical, biological, and conditional factors are incorporated into treatment algorithms. In contrast, no universally accepted resectability classification currently exists for PHC. We previously proposed an anatomical resectability classification based exclusively on biliary extension and vascular involvement and subsequently introduced NAC-GS for patients with BR and UR-LA disease and those with FDG-PET-positive regional LN metastasis [10,11]. The present study extends our previous reports by providing mature long-term outcome data with more than 5 years of follow-up in patients managed according to this treatment framework. Although our resectability classification intentionally focuses on anatomical factors, anatomical resectability alone may not fully capture the biological heterogeneity or physiological condition of patients with PHC. Future treatment algorithms may benefit from integrating anatomical factors with biological features, including serum CEA and CA19-9 levels, FDG-PET-based regional LN status, and response to NAC, as well as conditional factors such as performance status and functional remnant liver reserve. Such an “anatomy + biology + condition” framework may allow more individualized selection of candidates for NAC and conversion surgery and should be evaluated prospectively in larger multi-institutional cohorts.

Several retrospective studies have investigated NAC for PHC. Matsuyama et al. reported encouraging surgical outcomes after NAC-GS for BR-PHC using a different definition of borderline resectability. However, long-term survival beyond 5 years has not been reported, and evidence supporting standardized treatment strategies according to resectability remains limited. Our study extends these observations by demonstrating durable long-term outcomes after a standardized multidisciplinary treatment strategy applicable across all anatomical resectability categories.

Recent population-based studies using large national databases have increasingly suggested a survival benefit of NAC for BTC [24,25,26,27,28,29,30], particularly in patients with advanced-stage disease or regional LN metastasis [31]. Propensity score-matched analyses have demonstrated improved survival among selected patients with extrahepatic BTC receiving NAC, supporting a strategy of selective administration rather than routine use [31]. These findings are consistent with our treatment strategy, in which NAC was selectively administered to patients with anatomically advanced disease and/or FDG-PET-positive regional LN metastasis.

An important finding of the present study is that anatomical resectability was significantly associated with prognosis in the overall cohort. The resection rate decreased progressively from R to BR and UR-LA disease, indicating that anatomical resectability is closely associated with the likelihood of achieving curative-intent resection. In contrast, among patients who ultimately underwent curative-intent resection, no significant differences in OS or RFS were observed according to initial anatomical resectability. These findings support the prognostic value of anatomical resectability, particularly with respect to the likelihood of achieving curative-intent resection. However, they should not be interpreted as evidence that the multidisciplinary treatment strategy itself improved survival, because the present study did not include a contemporaneous control group managed with an alternative treatment strategy.

Among patients who ultimately underwent curative-intent operation, selected patients who received NAC-GS showed favorable long-term survival despite having more advanced disease characteristics than those who underwent upfront surgery. However, this finding should be interpreted with caution. The survival analysis included only patients who ultimately underwent curative-intent resection after NAC, thereby excluding those who did not proceed to surgery because of disease progression, insufficient remnant liver function, deterioration in performance status, or other reasons. Consequently, substantial selection bias is inherent in this comparison. In addition, the NAC and upfront surgery groups differed considerably in their baseline disease characteristics, reflecting the predefined treatment strategy in which NAC was preferentially administered to patients with anatomically advanced disease and/or clinically positive regional LN metastasis. Therefore, the present findings do not demonstrate equivalent efficacy between NAC and upfront surgery, nor do they establish a survival benefit of NAC. Rather, they indicate that favorable long-term outcomes can be achieved in selected patients with initially more advanced disease who successfully proceed to curative-intent operation after NAC.

The potential benefits of NAC-GS are likely multifactorial. In addition to improving local tumor control, NAC allows identification of occult metastatic disease during treatment, thereby avoiding unnecessary operation in patients with rapidly progressive disease. It also facilitates delivery of systemic chemotherapy before operation, when postoperative complications or impaired liver function may preclude adequate adjuvant treatment. Furthermore, NAC may increase the opportunity for conversion surgery in selected patients with initially UR-LA disease. In the present study, six patients initially classified as UR-LA underwent conversion surgery with curative intent after NAC. Although the small number of patients precludes definitive conclusions regarding survival benefit, these findings demonstrate the feasibility of conversion surgery in selected patients with initially anatomically unresectable disease.

The present study also provides important insight into the future direction of neoadjuvant treatment. In the multivariable analysis, elevated preoperative serum CEA and pathological T4 disease remained significantly associated with poorer survival after resection. However, given the limited number of outcome events relative to the number of variables included in the model, these findings should be interpreted cautiously and require validation in larger cohorts. Unlike anatomical factors, serum CEA may reflect biological tumor aggressiveness. Furthermore, in our exploratory pathological response analysis of 33 patients, no statistically significant differences in OS or RFS were observed between the high- and low-response groups, although high responders showed a numerical trend toward more favorable long-term outcomes. Given the small sample size and the absence of statistically significant survival differences, these findings should be regarded as hypothesis-generating and do not establish pathological response as a prognostic or surrogate marker. Further studies in larger cohorts are required to determine the potential clinical significance of pathological response after NAC.

Recent advances in systemic therapy are rapidly changing the treatment landscape of BTC. A recent multicenter randomized phase 2–3 trial demonstrated that neoadjuvant GOLP (gemcitabine, oxaliplatin, lenvatinib, and toripalimab) significantly prolonged event-free survival compared with upfront surgery in patients with resectable high-risk intrahepatic cholangiocarcinoma, providing the first high-level evidence supporting neoadjuvant therapy for BTC [32]. Although these findings cannot be directly extrapolated to PHC because of substantial differences in tumor biology, anatomical complexity, and surgical strategy, they strongly support the concept that biologically effective systemic therapy administered before surgery can improve long-term outcomes. In our cohort, patients with a favorable pathological response showed a numerical trend toward better survival; however, this exploratory observation was based on a small number of patients and did not reach statistical significance. Therefore, the present study does not establish pathological response as a surrogate marker of treatment efficacy, and its potential prognostic significance requires further investigation. Prospective randomized trials evaluating NAC for BTC are currently ongoing. In Japan, the multicenter phase III JCOG1920 (NABICAT) trial is evaluating NAC-GCS, whereas the international phase II/III PURITY trial is investigating neoadjuvant gemcitabine, cisplatin, and nab-paclitaxel (GAP) for patients with resectable BTC at high risk of recurrence [33]. Our institutional neoadjuvant strategy has already evolved from GS to GCS. Therefore, the present study provides an important benchmark representing the mature long-term outcomes achieved with GS-based neoadjuvant treatment. Future prospective studies should determine whether intensified neoadjuvant regimens can improve pathological response and long-term survival, particularly in patients with biologically aggressive disease.

This study has several limitations. First, this was a retrospective single-center study and was therefore subject to selection bias. Second, the relatively long study period from 2010 to 2021 may have introduced temporal changes in clinical practice, including perioperative management, adjuvant chemotherapy, and individualized additional preoperative treatment. These changes may have influenced treatment selection and long-term outcomes and should be considered when interpreting the present results. Third, the comparison between upfront surgery and NAC was not randomized, baseline disease severity differed substantially between the groups, and the survival analysis included only patients who ultimately underwent curative-intent operation. Therefore, this comparison was subject to substantial selection bias and cannot establish the comparative efficacy or survival benefit of NAC over upfront surgery. Fourth, the number of patients with UR-LA disease who ultimately underwent resection was relatively small. In addition, the multivariable Cox regression analysis included five variables based on 42 outcome events among 71 resected patients. Given the limited number of events relative to the number of variables included, the possibility of model overfitting and instability of the estimated effect sizes cannot be excluded. Therefore, the prognostic factors identified in this analysis should be considered exploratory and require validation in larger independent cohorts. Finally, initial resectability classification and post-NAC reassessment were based on multidisciplinary consensus, and formal interobserver agreement was not evaluated. Therefore, the reproducibility of the anatomical resectability classification and post-NAC reassessment requires external validation. Despite these limitations, the present study provides mature long-term outcome data that may serve as a benchmark for future prospective evaluation of resectability-based multidisciplinary treatment strategies for PHC.

## 5. Conclusions

The present study provides the first mature long-term evidence supporting a multidisciplinary treatment strategy based on anatomical resectability classification and clinical LN status using NAC-GS for PHC. In this single-center cohort, favorable long-term outcomes were observed in selected patients who ultimately underwent curative-intent resection, including patients who received NAC-GS for initially advanced disease. These findings provide feasibility and benchmark data for a resectability-based multidisciplinary treatment strategy but do not establish a survival benefit of NAC-GS over upfront surgery. Prospective multi-institutional studies are warranted to validate this treatment framework and to determine the therapeutic impact of NAC.

## Figures and Tables

**Table 1:** Resectability classification of localized perihilar cholangiocarcinoma from surgical points of view.

Criteria	Biliary Factor (Curative Resection Can Be Obtained by Either Side of Right or Left TSN or Less Hepatectomy)		Vascular Factor (Combined VR with Reconstruction of PV and/or HA)
**R**	Yes	and	Not required
**BR**	Yes	and	Required and technically feasible
**UR-LA**	No	and/or	Required but technically infeasible

TSN: trisectionectomy, VR: vascular resection, PV: portal vein, HA: hepatic artery, R: resectable, BR: borderline resectable, UR-LA: unresectable locally advanced.

**Table 2:** Baseline clinicopathological characteristics according to anatomical resectability classification.

Factors	R (*n* = 47)	BR (*n* = 38)	UR-LA (*n* = 25)	*p* Value
Age (years)	72 (44–87)	71.5 (50–84)	73 (45–85)	0.878
Gender (male/female)	26/21	24/14	18/7	0.374
Patients with biliary drainage	38 (80.9)	28 (73.7)	19 (76.0)	0.725
**Initial blood examination**				
Neutrophil-to-lymphocyte ratio	2.19 (0.97–8.15)	2.09 (1.09–7.90)	2.74 (0.77–10.59)	0.194
Prognostic nutritional index	43.4 (30.9–55.6)	45.1 (35.2–61.5)	43.0 (35.6–58.1)	0.489
CEA (ng/mL)	3.1 (0.7–38.4)	3.6 (1.1–16.3)	3.7 (1.6–65.7)	0.681
CA19-9 (U/mL)	94.4 (0.4–17,592)	150.0 (0.2–76,452)	233.8 (5.2–5265)	0.082
**Initial clinical staging**				
cT4 (%)	0	30 (78.9)	16 (64.0)	<0.001 *
Clinical LN metastasis	12 (25.2)	14 (36.8)	14 (56.0)	0.038 **
**Neoadjuvant GS therapy**	13 (27.7)	36 (94.7)	23 (92.0)	<0.001 *
Completion rate of initial 2 cycles	11 (84.6)	34 (94.4)	21 (91.3)	
Median relative dose intensity of S-1 (%)	100%	100%	100%	
Median relative dose intensity of G (%)	93.4%	93.3%	83.6%	
Patients with adverse events (%)	8 (61.5%)	20 (55.5%)	15 (65.2%)	
Toxicity grade 1/2	4	6	6	
Toxicity grade 3/4 (grade 3 cholangitis)	3 (2)	14 (7)	9 (5)	
**Resection**	40 (85.1)	25 (65.8)	6 (24.0)	<0.001 **

* R vs. BR, UR-LA; ** R, BR vs. UR-LA. R: resectable, BR: borderline resectable, UR-LA: unresectable locally advanced, CEA: carcinoembryonic antigen, CA19-9: carbohydrate antigen 19-9, LN: lymph node, GS: gemcitabine plus S-1. Data are expressed as number (percentage), median (range).

**Table 3:** Clinicopathological characteristics of patients undergoing curative-intent resection.

Factors	R (*n* = 40)	BR (*n* = 25)	UR-LA (*n* = 6)	*p* Value
**Age (years)**	71.5 (44–87)	70 (50–84)	64.5 (53–84)	0.463
**Gender (male/female)**	21/19	17/8	4/2	0.431
**Biliary drainage**	34 (85.0)	16 (64.0)	5 (83.3)	0.134
**Neoadjuvant therapy**	8 (20.0)	23 (92.0)	6 (100)	<0.001 *
**Chemotherapy**	8	23	4	
**Chemoradiotherapy**	0	0	2	
**Portal vein embolization (%)**	6 (15.0)	4 (16.0)	1 (16.7)	0.991
**Preoperative blood examination**				
**Neutrophil-to-lymphocyte ratio**	2.28 (0.84–8.15)	2.11 (0.63–6.15)	3.86 (0.99–6.46)	0.269
**Prognostic nutritional index**	43.4 (33.9–55.6)	45.9 (36.3–55.7)	42.6 (32.7–46.8)	0.235
**CEA (ng/mL)**	2.9 (0.5–30.8)	3.8 (1.0–24.4)	2.6 (2.2–5.4)	0.364
**CA19-9 (U/mL)**	59.6 (0.4–11,659)	60.8 (0.1–1158)	196.6 (8.0–669.3)	0.802
**Intraoperative outcomes**				
**Operation time (min)**	607 (404–943)	601 (383–805)	597 (395–663)	0.929
**Blood loss (mL)**	1385 (166–5166)	1459 (160–6059)	2384 (1443–4090)	0.126
Type of hepatectomy **				
**S1,2,3,4**	14	14	2	
**S1,2,3,4,5,8**	1	2	1	
**S1,5,6,7,8**	10	7	2	
**S1,4,5,6,7,8**			1	
**S1,4,5,8**	1	1		
**S1,5,8**	1			
**S1with HBR**	2			
**HBR**	2			
**Pancreatoduodenectomy with HBR**	6			
**Hepatopancreatoduodenectomy**	3	1		
**Combined vascular resection**	4 (10.0)	19 (76.0)	4 (66.7)	<0.001 *
**PV resection**	4 (10.0)	18 (72.0)	3 (50.0)	<0.001 *
**HA resection**	0 (0.0)	8 (32.0)	1 (16.7)	<0.001 *
**Morbidity and Mortality**				
**Clavien–Dindo ≥ III complication**	21 (52.5)	13 (52.0)	3 (50.0)	0.993
**Bile leakage, ISGLS grade B/C**	4 (10.0)	4 (16.0)	0	0.500
**PHLF, ISGLS grade B/C**	12 (30.0)	6 (24.0)	3 (50.0)	0.454
**Post-hepatectomy hemorrhage,** **ISGLS grade B/C**	2 (5.0)	1 (4.0)	0	0.849
**POPF, ISGPS grade B/C**	2 (5.0)	0	0	0.450
**Intra-abdominal abscess** **requiring drainage**	8 (20.0)	5 (20.0)	2 (33.3)	0.736
**Others**	6 (15.0)	5 (20.0)	0	0.473
**Reoperation**	3 (7.5)	1 (4.0)	1 (16.7)	0.545
**90-day mortality**	0	1 (4.0)	0	0.440
**Postoperative hospital stays (days)**	40.5 (19–161)	39 (13–136)	43 (13–115)	0.644
**Completed adjuvant chemotherapy**	20 (50.0)	19 (76.0)	4 (66.7)	0.108
**Pathological finding**				
**Histological differentiation**				0.473
**G1/G2/G3/MANEC**	31/7/1/1	21/2/2/0	4/2/0/0	0.596
**pT4**	2 (5.0)	8 (32.0)	3 (50.0)	0.003 *
**Lymph node metastasis**	17 (42.5)	13 (52.0)	3 (50.0)	0.744
**Intrahepatic metastasis**	1 (2.5)	2 (8.0)	0	0.487
**Residual tumor**				
**R0/R1/R2**	29 (72.5)/6/5	16 (64.0)/7/2	4 (66.7)/1/1	0.743
**pHM0/pHM1/pHM2**	31/7/2	23/2/0	5/0/1	0.229
**pDM0/pDM1/pDM2**	38/1/1	24/0/1	6/0/0	0.895
**pEM0/pEM1/pEM2**	35/4/1	17/7/1	4/2/0	0.319

* R vs. BR, UR. R: resectable, BR: borderline resectable, UR-LA: unresectable locally advanced, CEA: carcinoembryonic antigen, CA19-9: carbohydrate antigen 19-9, HBR: hilar bile duct resection, PV: portal vein, HA: hepatic artery, ISGLS: International Study Group for Liver Surgery, PHLF: Post-hepatectomy liver failure, POPF: postoperative pancreatic fistula, ISGPS: International Study Group for Pancreatic Surgery, MANEC: mixed adenoneuroendocrine carcinoma, R0: complete resection, R1: microscopic residual tumor resection, R2: macroscopic residual tumor resection or distant metastasis (intrahepatic metastasis), pHM: proximal bile duct margin, pDM: distal bile duct margin, pEM: dissected margin. Data are expressed as number (percentage), median (range). ** Expressed as Couinaud’s hepatic segments resected.

**Table 4:** Comparison of characteristics between up-front surgery and NAC followed by surgery.

Factors	Up Front Surgery (*n* = 34)	NAC Followed by Surgery (*n* = 37)	*p* Value
**Age (years)**	71.5 (44–87)	70.0 (50–87)	0.600
**Gender (male/female)**	18/16	24/13	0.342
**Biliary drainage**	28 (82.4)	27 (73.0)	0.403
**Neoadjuvant therapy**	–	37	
**Chemotherapy**	–	35	
**Chemoradiotherapy**	–	2	
**Portal vein embolization (%)**	5 (14.7%)	6 (16.2%)	1.000
**Preoperative blood examination**			
**Neutrophil-to-lymphocyte ratio**	2.26 (0.88–8.15)	3.26 (0.63–6.46)	0.615
**Prognostic nutritional index**	43.8 (33.4–55.6)	43.7 (32.7–54.6)	0.941
**CEA (ng/mL)**	2.9 (0.5–30.8)	2.6 (2.2–5.4)	0.183
**CA19-9 (U/mL)**	59.6 (0.4–1980)	69.7 (0.1–11,659)	0.945
**Intraoperative outcomes**			
**Operation time (min)**	608 (404–943)	600 (383–805)	0.561
**Blood loss (mL)**	1341 (166–5166)	2037 (160–6059)	0.136
**Type of hepatectomy** ***			
**S1,2,3,4**	12	18	
**S1,2,3,4,5,8**	1	2	
**S1,5,6,7,8**	9	11	
**S1,4,5,6,7,8**		1	
**S1,4,5,8**	1	1	
**S1,5,8**	1		
**S1with HBR**	2		
**HBR**		2	
**Pancreatoduodenectomy with HBR**	5	1	
**Hepatopancreatoduodenectomy**	3	1	
**Combined vascular resection**	6 (17.6)	21 (56.8)	**0.001**
**PV resection**	6 (17.6)	19 (51.4)	**0.006**
**HA resection**	1 (2.9)	8 (21.6)	**0.029**
**Morbidity and Mortality**			
**Clavien–Dindo ≥ III complication**	17 (50.0)	20 (54.1)	0.814
**90-day mortality**	0	1 (2.7)	1.000
**Postoperative hospital stays (days)**	39 (22–146)	40 (13–161)	0.886
**Completed adjuvant chemotherapy**	18 (52.9%)	25 (67.6%)	0.233
**Pathological finding**			
**Histological differentiation**			
**G1/G2/G3/MANEC**	27/6/1/0	29/5/2/1	0.712
**pT4**	3 (8.8)	10 (27.0)	0.066
**Lymph node metastasis**	16 (47.1)	17 (45.9)	1.000
**Intrahepatic metastasis**	1 (2.9)	1 (2.7)	1.000
**Residual tumor**			
**R0/R1/R2**	26 (76.5)/5/3	23 (62.2)/9/5	0.427

NAC: neoadjuvant chemotherapy, CEA: carcinoembryonic antigen, CA19-9: carbohydrate antigen 19-9, R0: complete resection, HBR: hilar bile duct resection, PV: portal vein, HA: hepatic artery, *** Expressed as Couinaud’s hepatic segments resected, PV: portal vein, HA: hepatic artery, MANEC: mixed adenoneuroendocrine carcinoma, R1: microscopic residual tumor resection, R2: macroscopic residual tumor resection or distant metastasis (intrahepatic metastasis). Data are expressed as number (percentage), median (range).

**Table 5:** Univariable and multivariable analyses of prognostic factors for overall survival after curative-intent resection.

Factors	Univariate Analysis				Multivariate Analysis	
	No. of Patients	5-Year Survival Rate (%)	Median Survival Time (Month)	*p* Value	Hazard Ratio (95% CI)	*p* Value
**Preoperative CA19-9**						
**<37 U/mL**	28	50.0	-	0.216		
**37 U/mL** **≤**	43	40.6	47.1			
**Preoperative CEA**						
**<5.5 ng/mL**	58	51.7	74.0	**<0.001**	3.174 (1.496–6.737)	**0.003**
**5.5 ng/mL** **≤**	13	17.6	21.2			
**Neoadjuvant therapy**						
**not done**	34	41.2	53.8	0.744		
**done**	37	50.3	60.8			
**Adjuvant therapy**						
**not done**	21	23.8	37.3	**0.026**	2.222 (1.117–4.424)	**0.023**
**done**	50	53.1	78.9			
**Combined vascular resection**						
**not done**	44	40.9	53.8	0.772		
**done**	27	54.3	74.0			
**pT factor (AJCC 8th)**						
**≤** **pT3**	58	50.0	60.8	0.071	2.397 (1.053–5.460)	**0.037**
**pT4** **≤**	13	26.4	31.4			
**Lymph node metastasis**						
**Negative**	38	57.0	57.0	0.156		
**Positive**	33	44.0	44.0			
**Intrahepatic metastasis**						
**Absent**	68	47.1	60.8	**0.017**	2.597 (0.502–13.425)	0.255
**Present**	3	-	-			
**Curative resection**						
**R0**	49	53.1	-	**0.019**	1.755 (0.918–3.3569)	0.089
**R1, 2**	22	28.7	37.3			

CEA: carcinoembryonic antigen, CA19-9: carbohydrate antigen 19-9, R0: complete resection, R1: microscopic residual tumor resection, R2: macroscopic residual tumor resection or distant metastasis (intrahepatic metastasis).

## Data Availability

The datasets generated and/or analyzed during the current study are available from the corresponding author on reasonable request.

## Supplementary Materials

The following supporting information can be downloaded at: https://www.mdpi.com/article/10.3390/cancers18193238/s1, Supplementary Figure S1. Pathological response to gemcitabine plus S-1-based neoadjuvant chemotherapy. Representative pathological findings of (a) low responders (≥30% viable tumor cells) and high responders (<30% viable tumor cells) according to the proportion of residual viable cancer cells in the resected specimen. Supplementary Figure S2. Overall survival and recurrence-free survival curves between low responder and high responder patients. (a) Overall survival, (b) recurrence-free survival. Supplementary Table S1. Comparison of characteristics between pathological low and high responder.

## Abbreviations

The following abbreviations are used in this manuscript: AC, adjuvant chemotherapy; AJCC, American Joint Committee on Cancer; BR, borderline resectable; BTC, biliary tract cancer; CA19-9, carbohydrate antigen 19-9; CEA, carcinoembryonic antigen; CI, confidence interval; CRT, chemoradiation therapy; CTCAE, Common Terminology Criteria for Adverse Events; DSS, disease-specific survival; FDG-PET, fluorodeoxyglucose positron emission tomography; GAP, gemcitabine, cisplatin, and nab-paclitaxel; GCS, gemcitabine, cisplatin, and S-1; GEM, gemcitabine; GOLP, gemcitabine, oxaliplatin, lenvatinib, and toripalimab; GS, gemcitabine plus S-1; HA, hepatic artery; HPD, hepatopancreatoduodenectomy; HR, hazard ratio; LN, lymph node; MST, median survival time; NAC, neoadjuvant chemotherapy; NAC-GS, neoadjuvant chemotherapy with gemcitabine plus S-1; OS, overall survival; PHC, perihilar cholangiocarcinoma; PV, portal vein; R, resectable; RDI, relative dose intensity; RFS, recurrence-free survival; UR-LA, unresectable locally advanced.
