# Supplementary material for: Long-Term Outcomes of a Multidisciplinary Treatment Strategy Using Neoadjuvant Gemcitabine Plus S-1 Based on Anatomical Resectability Classification and Lymph Node Status in Patients with Perihilar Cholangiocarcinoma
Source: Cancers (Basel). 2026 Oct 8;18(19):3238. doi: 10.3390/cancers18193238 (PMC13664265; doi:10.3390/cancers18193238)
Supplement: Supplementary file 1 [file cancers-18-03238-s001.zip › Supplementary Table S1.pdf]

Supplementary Table S1. Comparison of characteristics between pathological low and high responder.

Factors	Low responder (n=13)	High responder (n=20)	*p* value
Age (years)	71.5 (44-87)	70.0 (50-87)	0.600
Gender (male / female)	4 / 9	6 / 14	1.000
Biliary drainage	9 (69.2%)	14 (70.0%)	1.000
Adjuvant therapy	13	20	
Chemotherapy	13	18	
Chemoradiotherapy	0	2	
Portal vein embolization (%)	1 (7.7%)	3 (15.0%)	1.000
**Preoperative blood examination**			
Neutrophil-to-lymphocyte ratio	2.03 (0.73-6.46)	2.26 (0.63-5.06)	0.819
Prognostic nutritional index	45.5 (33.4-54.6)	45.2 (32.7-52.0)	0.662
CEA (ng/ml)	3.7 (1.7-9.6)	3.4 (1.0-24.4)	0.842
CA19-9 (U/ml)	60.8 (0.1-11,659)	78.2 (0.6-1,158)	0.676
**Intraoperative outcomes**			
Operation time (min)	535 (383-805)	604 (395-681)	0.169
Blood loss (ml)	2,367 (719-4,103)	1,632 (160-6,059)	0.548
**Type of hepatectomy***			
S1,2,3,4	7	11	
S1,2,3,4,5,8		2	
S1,5,6,7,8	4	5	
S1,4,5,6,7,8		1	
S1,4,5,8	1		
HBR	1	1	
**Combined vascular resection**	7 (53.8)	14 (70.0)	0.465
PV resection	7 (53.8)	12 (60.0)	1.000
HA resection	2 (15.4)	6 (30.0)	0.431
**Morbidity and Mortality**			
Clavien-Dindo≥III complication	6 (46.2)	13 (65.0)	0.472
90-day mortality	1 (7.7)	0	0.394
**Postoperative hospital stays (days)**	40 (13-115)	41.5 (13-161)	0.730
Completed adjuvant chemotherapy	8 (61.5%)	16 (80.0%)	0.425
**Histological differentiation**			0.473
G1 / G2 / G3	10 / 2 / 1	16 / 3 / 1	0.949
**Pathological finding**			
pT4	4 (30.8%)	6 (30.0%)	1.000

Lymph node metastasis	8 (61.5%)	7 (35.0%)	0.169
Intrahepatic metastasis	1 (7.7%)	1 (5.0%)	1.000
**Residual tumor**			
R0 / R1 / R2	8 (61.5) / 3 / 2	13 (65.0) / 5 / 2	0.898

CEA: carcinoembryonic antigen, *CA19-9*: carbohydrate antigen 19-9, *R0*: complete resection, *HBR*: hilar bile duct resection, *PV*: portal vein, *HA*: hepatic artery

∗Expressed as Couinaud's hepatic segments resected, PV: portal vein, HA: hepatic artery

R1: microscopic residual tumor resection, *R2*: macroscopic residual tumor resection or distant metastasis (intrahepatic metastasis), Data are expressed as number (percentage), median (range)
